# Distant metastasis prediction via a multi-feature fusion model in breast cancer

**DOI:** 10.18632/aging.103630

**Published:** 2020-09-28

**Authors:** Wenjuan Ma, Xin Wang, Guijun Xu, Zheng Liu, Zhuming Yin, Yao Xu, Haixiao Wu, Vladimir P. Baklaushev, Karl Peltzer, Henian Sun, Natalia V. Kharchenko, Lisha Qi, Min Mao, Yanbo Li, Peifang Liu, Vladimir P. Chekhonin, Chao Zhang

**Affiliations:** 1 Department of Breast Imaging, Tianjin Medical University Cancer Institute and Hospital, National Clinical Research Center for Cancer, Key Laboratory of Cancer Prevention and Therapy, Tianjin’s Clinical Research Center for Cancer, Tianjin 300060, China; 2 Department of Epidemiology and Biostatistics, First Affiliated Hospital, Army Medical University, Chongqing 400038, China; 3 Department of Bone and Soft Tissue Tumors, Tianjin Medical University Cancer Institute and Hospital, National Clinical Research Center for Cancer, Key Laboratory of Cancer Prevention and Therapy, Tianjin’s Clinical Research Center for Cancer, Tianjin 300060, China; 4 Department of Breast Oncoplastic Surgery, Tianjin Medical University Cancer Institute and Hospital, National Clinical Research Center for Cancer, Key Laboratory of Breast Cancer Prevention and Therapy, Tianjin Medical University, Ministry of Education, Key Laboratory of Cancer Prevention and Therapy, Tianjin, Tianjin’s Clinical Research Center for Cancer, Sino-Russian Joint Research Center for Oncoplastic Breast Surgery, Tianjin 300060, China; 5 Federal Research and Clinical Center of Specialized Medical Care and Medical Technologies, Federal Biomedical Agency of the Russian Federation, Moscow 115682, Russian Federation; 6 Department of Research and Innovation, University of Limpopo, Turfloop 0527, South Africa; 7 Department of Oncology, N.N. Blokhin National Medical Research Center of Oncology, Moscow 115478, Russian Federation; 8 Department of Oncology, Radiology and Nuclear Medicine, Medical Institute of Peoples’ Friendship University of Russia, Moscow 117198, Russian Federation; 9 Department of Pathology, Tianjin Medical University Cancer Institute and Hospital, National Clinical Research Center for Cancer, Key Laboratory of Cancer Prevention and Therapy, Tianjin, Tianjin’s Clinical Research Center for Cancer, Tianjin 300060, China; 10 Department of Pathology and Southwest Cancer Center, First Affiliated Hospital, Army Medical University, Chongqing 400038, China; 11 Department of Basic and Applied Neurobiology, Federal Medical Research Center for Psychiatry and Narcology, Moscow 117997, Russian Federation

**Keywords:** breast neoplasms, neoplasm metastasis, early detection, artificial intelligence

## Abstract

This study aimed to develop a model that fused multiple features (multi-feature fusion model) for predicting metachronous distant metastasis (DM) in breast cancer (BC) based on clinicopathological characteristics and magnetic resonance imaging (MRI). A nomogram based on clinicopathological features (clinicopathological-feature model) and a nomogram based on the multi-feature fusion model were constructed based on BC patients with DM (n=67) and matched patients (n=134) without DM. DM was diagnosed on average (17.31±13.12) months after diagnosis. The clinicopathological-feature model included seven features: reproductive history, lymph node metastasis, estrogen receptor status, progesterone receptor status, CA153, CEA, and endocrine therapy. The multi-feature fusion model included the same features and an additional three MRI features (multiple masses, fat-saturated T2WI signal, and mass size). The multi-feature fusion model was relatively better at predicting DM. The sensitivity, specificity, diagnostic accuracy and AUC of the multi-feature fusion model were 0.746 (95% CI: 0.623-0.841), 0.806 (0.727-0.867), 0.786 (0.723-0.841), and 0.854 (0.798-0.911), respectively. Both internal and external validations suggested good generalizability of the multi-feature fusion model to the clinic. The incorporation of MRI factors significantly improved the specificity and sensitivity of the nomogram. The constructed multi-feature fusion nomogram may guide DM screening and the implementation of prophylactic treatment for BC.

## INTRODUCTION

Breast cancer (BC) is the most common malignant tumour in females worldwide and results in the highest mortality rate in women [1]. Distant metastasis (DM) remains the main cause of death in BC patients [2]. Over 30% of BC patients present with DM, which significantly worsens the prognosis [3, 4]. The National Comprehensive Cancer Network (NCCN) guidelines recommend that all BC patients be followed up every three months. Patients showing BC recurrence signs and metastatic symptoms should be screened for DM. Frequent screening may result in unnecessary radiation exposure and an economic burden for BC patients. Thus, prediction modelling of DM in BC is warranted.

A series of studies reported that patient age and past medical history were risk factors for DM in cancer patients [4–6]. Several pathological characteristics, including estrogen receptor (ER) status, progesterone receptor (PR) status, N stage, and histological differentiation, were reported to be associated with the occurrence of DM in BC [7–9]. However, due to the limited variability of the included clinicopathological characteristics, the reported predictive system could hardly make satisfactory predictions.

Magnetic resonance imaging (MRI) can comprehensively evaluate the overall tumour details of BC. It can also determine the heterogeneity of tumours by detecting haemodynamic characteristics and morphology [10]. MRI has been widely used to predict the prognosis of BC [11, 12]. Few studies have been conducted to predict DM in BC with a model that fuses multiple features (multi-feature fusion model).

Based on identified variables, the present study aimed to develop a multi-feature fusion model incorporating clinicopathological characteristics and MRI features for predicting metachronous DM in BC. The nomogram can potentially guide metachronous DM screening and the implementation of personalized therapy.

## RESULTS

### Characteristics of distant metastasis

**Figure 1:**
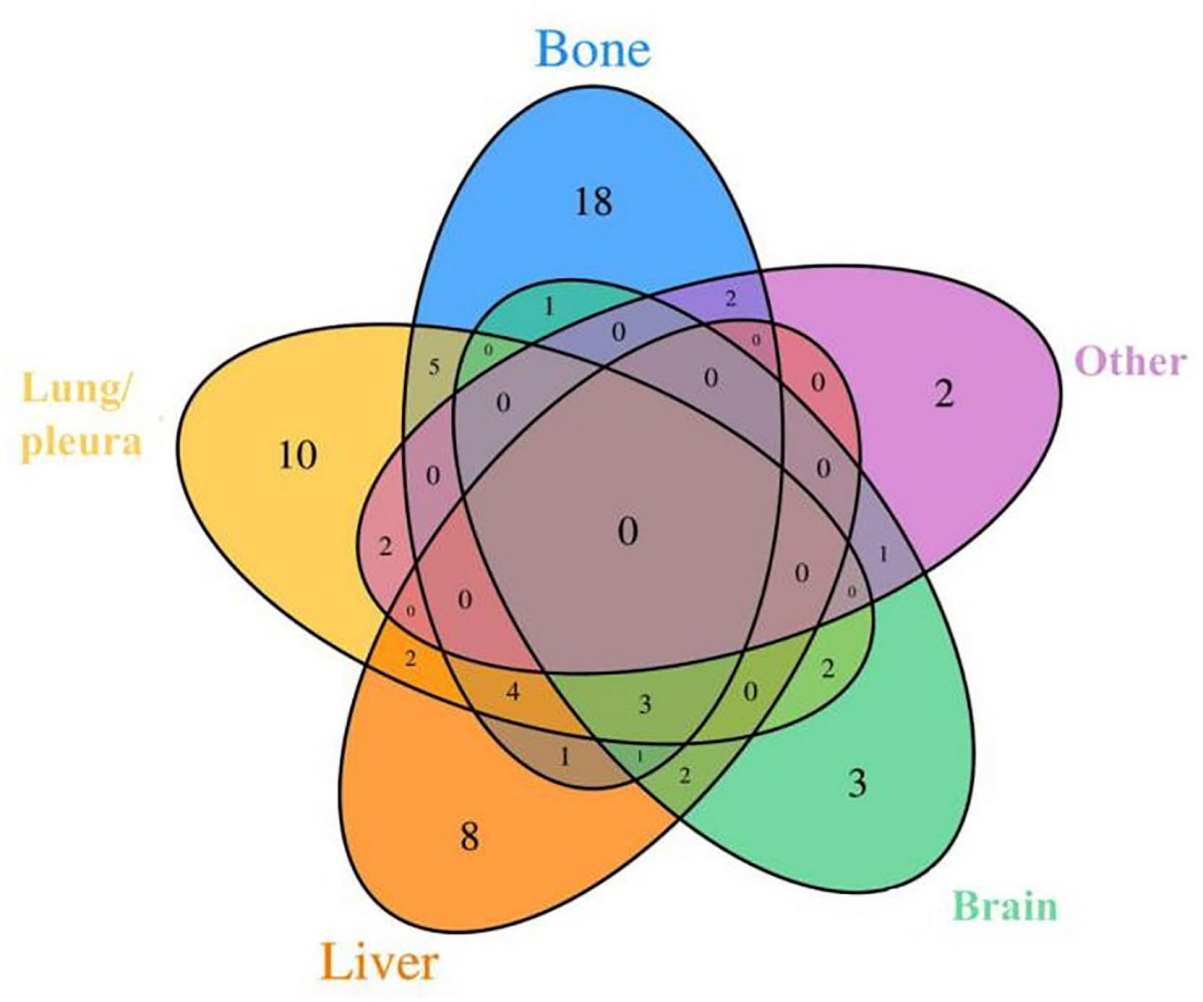
**Venn diagrams showing intersections between different metastasis types used in our study.** There were 26 cases of multiple organ metastases and 41 cases of single organ metastasis. Others include peritoneal (or mediastinal, ovarian, soft tissue) metastasis, pericardial effusion and lemostenosis.

The DM cohort included 41 patients with single organ metastasis and 26 patients with multiple organ metastases. Bone was the most common site for DM (35 cases, 52.24%), followed by the lung and/or pleura (28 cases, 41.79%) and liver (21 cases 31.34%). Visceral (hepatic, pulmonary, esophageal, and ovarian) metastases were found in 83.58% (56 cases) of patients. A Venn diagram was used to demonstrate the details of BC patients with different metastatic sites (Figure 1). The average period until the occurrence of DM after a BC diagnosis was 17.31±13.12 months.

### Differences in the clinicopathological and MRI features between BC patients with/without DM

Detailed information on the clinicopathological characteristics is shown in Supplementary Tables 1, 2. There was a significant difference in reproductive history (85.07% versus 95.52%, *χ^2^* =6.655; *p*=0.01), parity (*χ^2^* =21.860; p<0.001), metastatic lymph nodes (59.70% versus 24.63%, *χ^2^* =23.759; p<0.001), ER status (61.19% versus 79.85%, *χ^2^* =8.008; *p*=0.005), PR status (56.72% versus 77.61%, *χ^2^* =9.405; *p*=0.002), CA153 (25.37% versus 0.75%, *χ^2^* =33.225; *p*<0.001), CEA (19.40% versus 0.75%, *χ^2^* =23.993; *p*=<0.001), CA125 (14.62% versus 5.97%, *χ^2^* =5.696; *p*=0.017), surgery (*χ^2^* =19.168; *p*<0.001) and endocrine therapy (0.00% versus 8.96%, *χ^2^* =6.381; *p*=0.011) between patients with/without DM. There were no significant differences in the age distribution, family history of BC, marital status, number of abortions, age of menarche, HER2 status or Ki-67 expression, radiotherapy, or chemotherapy between the control group and metastatic group.

As shown in Supplementary Table 1, multiple masses (***χ^2^***=25.441; *p*<0.001), T1WI signal (***χ^2^***=8.127; *p*=0.004), fat-saturated T2WI signal (***χ^2^***=4.043; *p*=0.044), lesion size (***χ^2^***=31.855; *p*<0.001) and lesion type (***χ^2^***=10.090; *p*=0.006) were markedly different between these groups. No significant differences were found in the kinetic curve pattern, internal enhancement, parenchymal enhancement, or fibroglandular tissue between the control group and the metastatic group.

### Dimensionality reduction and feature selection

**Figure 2:**
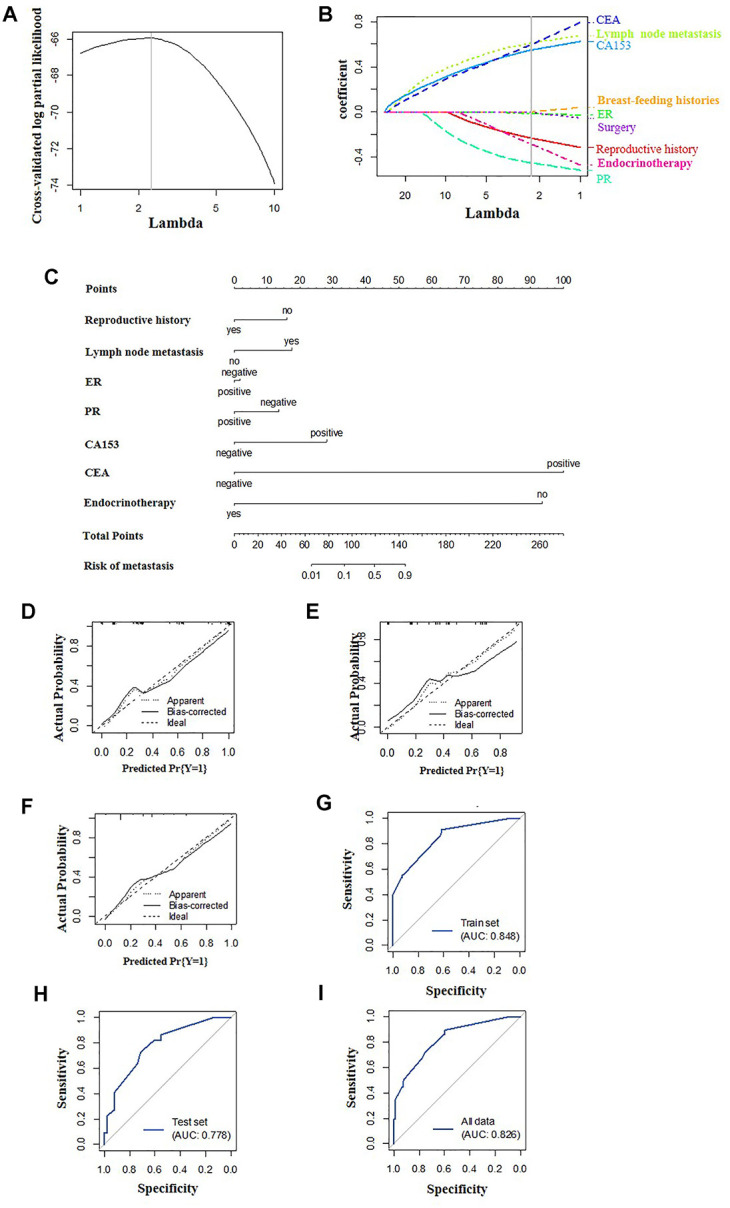
**Construction of the clinicopathological-feature alone model.** (**A**) Selection of tuning parameter lambda in the LASSO model used 10-fold cross-validation. The gray line in the figure is the partial likelihood estimate corresponding to the optimal value of lambda. The optimal lambda value of 2.313 was chosen. (**B**) LASSO coefficient profiles of the eleven selected features. A vertical line was plotted at the optimal lambda value, which resulted in seven features with nonzero coefficients. (**C**) A nomogram was developed in the training data set with clinicopathological characteristics. Calibration curves and ROC curves of the nomogram for the training set (**D**, **G**), validation set (**E**, **H**) and total population (**F**, **I**).

**Figure 3:**
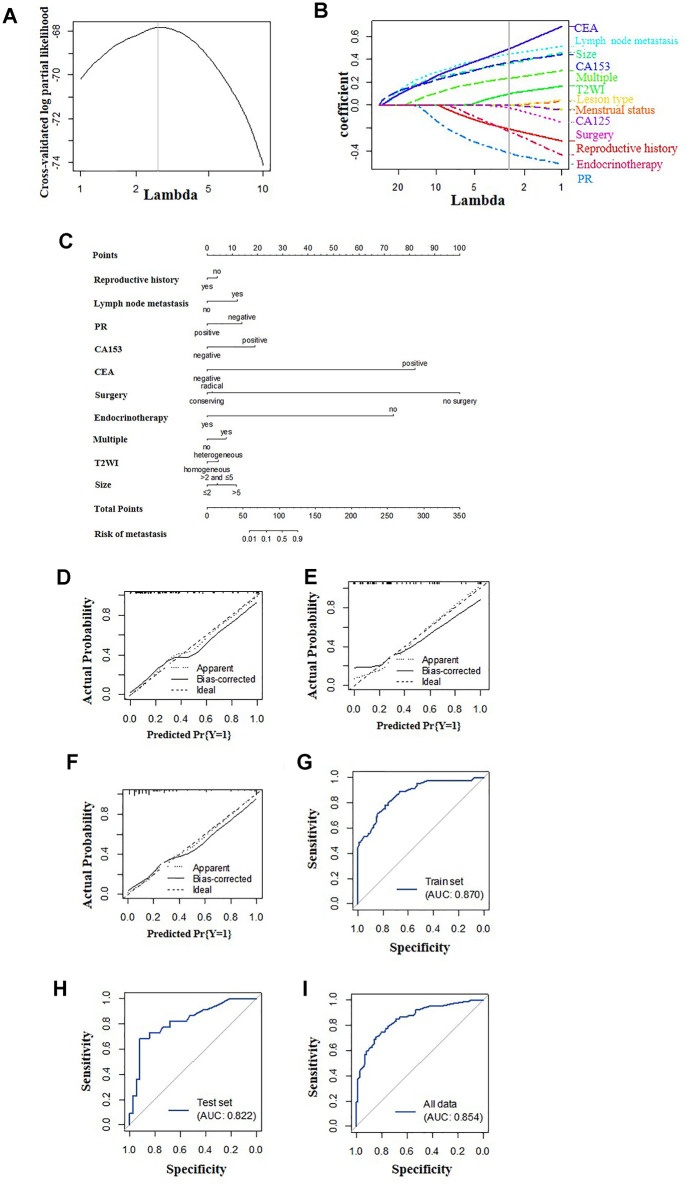
**Construction of the multi-feature fusion model.** (**A**) Selection of tuning parameter lambda in the LASSO model used 10-fold cross-validation. The gray line in the figure is the partial likelihood estimate corresponding to the optimal value of lambda. The optimal lambda value of 2.653 was chosen. (**B**) LASSO coefficient profiles of the sixteen selected features. A vertical line was plotted at the optimal lambda value, which resulted in ten features with nonzero coefficients. (**C**) A nomogram was developed in the training data set with clinicopathological and MRI features. Calibration curves and ROC curves of the nomogram for the training set (**D**, **G**), validation set (**E**, **H**) and total population (**F**, **I**).

According to the LASSO method, seven features with optimal λ values, including reproductive history, lymph node metastasis, ER status, PR status, CA153, CEA and endocrine therapy, were selected for the model with only clinicopathological features (clinicopathological-feature alone model) (Figure 2A, 2B). Ten features incorporating seven clinical features (reproductive history, lymph node metastasis, PR, CA153, CEA, surgery and endocrine therapy) and three MRI features (multiple masses, fat-saturated T2WI signal and mass size) were selected for the multi-feature fusion model (Figure 3A, 3B).

### Construction and validation of the predictive nomogram

Multivariate logistic regression was used to construct two models to predict DM in BC using the aforementioned features, the clinicopathological-feature alone model (Figure 2C) and the multi-feature fusion model (Figure 3C).

**Figure 4:**
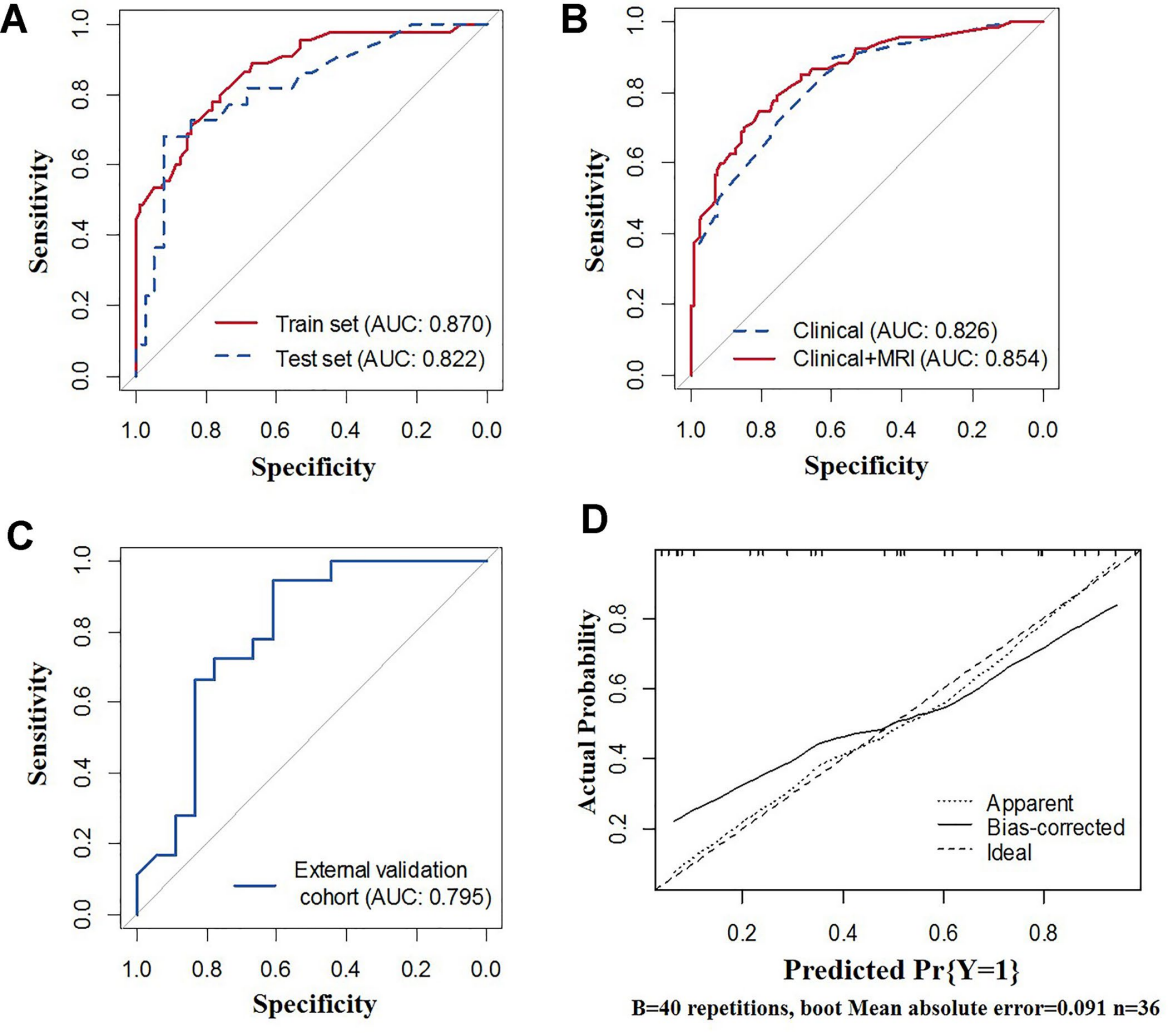
**Receiver operating characteristic (ROC) curves of the nomograms.** (**A**) ROC curves of the clinicopathological-feature alone model and multi-feature fusion model for the total population. (**B**) ROC curves of the multi-feature fusion model in the training set and calibration set. (**C**) ROC curve of the multi-feature fusion model in the external validation cohort. (**D**) Calibration curves of the multi-feature fusion model in the external validation cohort.

The calibration curve showed that the prediction (solid line) of the two models closely followed the 45-degree line in the training and test sets, suggesting good diagnostic accuracy (Figure 2D–2I for the clinicopathological-feature alone model and Figure 3D–3I for the multi-feature fusion model). The ROC curves of the clinicopathological-feature alone model showed AUCs of 0.848 (95% CI 0.780-0.915) and 0.778 (95% CI 0.660-0.896) in the training set and test set, respectively, and no significant difference was found between these values, indicating the reliability of the nomogram (D=1.003; *p*=0.318). The ROC curves of the multi-feature fusion model showed AUCs of 0.870 (95% CI 0.807-0.934) and 0.822 (95% CI 0.708-0.936) in the training set and test set, respectively, and no significant difference was found between these values, indicating the reliability of the nomogram (D=0.730; *p*=0.467) (Figure 4A).

### Differences in the prediction performance between the clinicopathological-feature alone model and the multi-feature fusion model

As shown in Figure 4B, the sensitivity, specificity, diagnostic accuracy and AUC of the predictive nomogram based on the clinicopathological-feature alone model were 0.896 (95% CI: 0.791-0.953), 0.597 (95% CI: 0.509-0.680), 0.697 (95% CI: 0.628-0.759), and 0.826 (95% CI: 0.759-0.882), respectively. The sensitivity, specificity, diagnostic accuracy and AUC of the predictive nomogram based on the multi-feature fusion model were 0.746 (95% CI: 0.623-0.841), 0.806 (95% CI: 0.727-0.867), 0.786 (95% CI: 0.723-0.841), and 0.854 (95% CI: 0.798-0.911), respectively. The multi-feature fusion model showed a relatively better performance than the clinicopathological-feature alone model (IDI=0.061, 95% CI: 0.029-0.094, *p*=0.002; D=1.451, *p*=0.147).

### External validation of the multi-feature fusion model

Detailed information on the clinicopathological and MRI characteristics of the external validation cohort is shown in Supplementary Table 1. As shown in Figure 4C, 4D, the sensitivity, specificity, diagnostic accuracy and AUC of the predictive nomogram based on the multi-feature fusion model were 0.708 (95% CI: 0.487-0.866), 0.917 (95% CI: 0.598-0.996), 0.778 (95% CI: 0.609-0.899) and 0.795 (95% CI: 0.640-0.949), respectively. The calibration curve showed that the prediction (solid line) of the multi-feature fusion model closely followed the 45-degree line in the external validation datasets, suggesting good generalizability of the prediction model.

## DISCUSSION

Distant metastasis represents the main reason for morbidity and mortality in BC patients. Approximately 7.15% of BC patients present with DM at diagnosis [8]. The most common metastatic sites are the bone, brain, liver, and lung [8]. Although several predictive models for DM in BC have been recently reported, a multi-feature fusion model can have better predictive ability [9, 13, 14].

Our nomogram was created with a combination of univariate analysis and the LASSO method. Our model is an improvement of previous predictive models based on univariate and multivariate analyses and optimized the predictive ability and stability (Figure 3). Using the LASSO method, ten characteristics were selected, including seven clinicopathological features and three MRI features. In the present study, we performed a comparative analysis between nomograms generated with/without MRI features. As shown in Figures 2 and 3, the incorporation of MRI features can significantly improve both the specificity and sensitivity of the predictive nomogram. Thus, to address the limitations of current nomograms merely generated with clinical factors, the incorporation of imaging data is important. Among all radiographic imaging methods, MRI was chosen for its ability to obtain mass information and its wide acceptance.

Previous studies demonstrated the potential ability of DCE-MRI to distinguish patients with/without metastasis [15]. It was reported that type 3 TIC patterns showed a significant association with the occurrence of DM. Our results showed that the enhancement pattern and TIC pattern were not significantly correlated with DM. This inconsistency might be caused by different proportions of the two factors between the present study and the previous study (only 6 of 59 patients had DM) [15]. Among the recorded MRI features, multiple masses, fat-saturated T2WI signal, and mass size were found to be independent predictors for DM in BC. A previous study reported tumour size as one of the risk factors for DM in BC [16]. We further verified this hypothesis through MRI features in BC patients with metachronous DM. A series of studies previously reported the different risk factors and prognostic factors of synchronous metastasis and metachronous metastasis in cancer [17, 18]. Thus, studies predicting DM should separate synchronous metastasis and metachronous metastasis.

Some independent clinicopathological factors were previously confirmed to be associated with DM. Age, T stage, N stage, lymphovascular invasion, and hormone receptor status were independently associated with bone metastasis in BC [19]. Moreover, histological subtypes and tumour grade have been reported to be significantly related to visceral metastasis in BC patients [20]. The latest study found that sex, histology type, N stage, grade, age, ER status, PR status, and HER2 status can predict liver metastasis in BC [9]. In our study, reproductive history, lymph node metastasis, PR status, CA153, CEA, surgery and endocrine therapy were found to be correlated with DM occurrence in BC. Although DM differed among patients with various molecular subtypes, the molecular subtype was not confirmed to be a significant factor. The underlying reasons will be clarified with a larger sample size.

This is the first study to construct a multi-feature fusion model for BC patients with metachronous DM. We admit to several limitations. First, considering the many subtypes of BC, the risk factors identified with the limited sample size may not be equally relevant in the general population. A larger multicentric validation will be needed. Second, some patients were followed up for less than 5 years. The rate of DM may be underestimated. Third, further studies will be needed to analyse the effect of incorporating other imaging data in the predictive nomogram, such as mammography and breast ultrasounds. Fourth, the present study merely included general features in the prediction model, and some features may not be captured; thus, the diagnostic accuracy of the prediction model was not satisfactory. In the future, radiomics is needed to incorporate more features into the model and improve the performance of the prediction model.

In summary, the characteristics of metachronous DM in BC were described and analysed. The average period until the occurrence of DM after a BC diagnosis was 17.31±13.12 months. Using an artificial intelligence technique, the dimensionality of imaging characteristics can be reduced and merged into the predictive nomogram for DM in BC. The constructed nomogram can potentially be employed as a graphic tool to guide metachronous DM screening and generate individualized treatment plans in BC.

## MATERIALS AND METHODS

### Study design and participants

**Figure 5:**
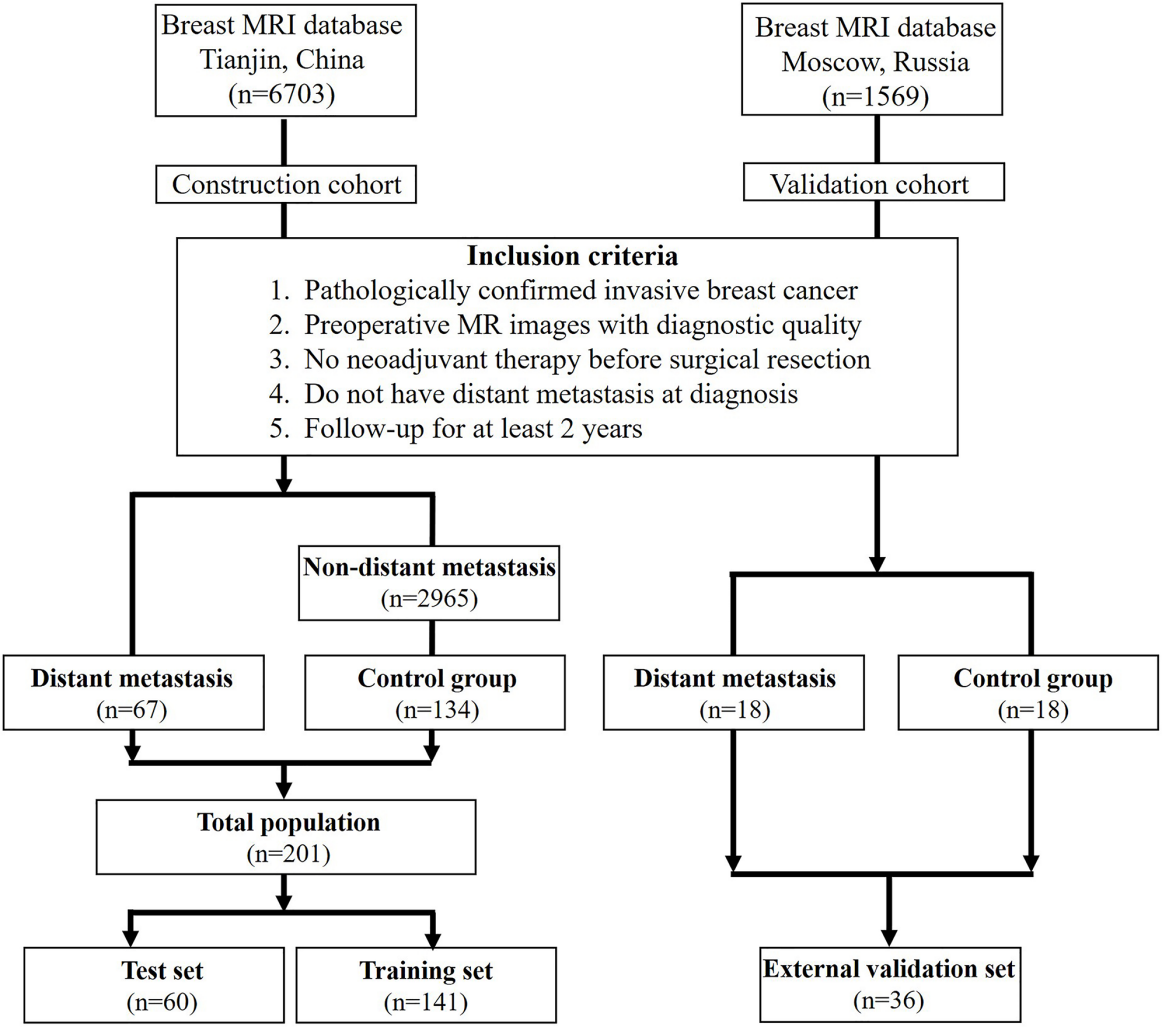
**Flowchart of the patient selection process in the present study.**

This case-control study was approved by the Ethics Committee of Tianjin Medical University Cancer Institute and Hospital. A total of 6,703 BC patients from January 2011 to December 2016 were reviewed from the database. The inclusion criteria were as follows (Figure 5): (1) a histopathological diagnosis of invasive BC through surgically resected specimens and/or needle biopsy; (2) availability of diagnostic-quality preoperative MRI images; (3) MRI scanning before neoadjuvant therapy or surgical resection; (4) no DM at diagnosis; and (5) follow-up data for at least two years. Eventually, sixty-seven patients (8 diagnosed by puncture biopsy, 3 by surgery, and 56 by imaging) with DM and 134 randomly selected patients without DM were included in the present study as the model construction cohort.

A series of demographic and clinicopathological characteristics were collected, including age, family history, breastfeeding history, marital status, abortion history, number of abortions, reproductive history, parity, menstrual status, age of menarche, clinical-based lymph node metastasis, estrogen receptor (ER) status, progesterone receptor (PR) status, human epidermal growth factor receptor 2 (HER2) status, Ki-67 expression, surgery, radiotherapy, and chemotherapy. According to the NCCN guidelines, breast cancer patients who received breast-conserving surgery (lumpectomy) with or without radiotherapy were recommended to receive endocrine therapy to reduce the risk of recurrence. Thus, endocrine therapy was also added as one of the potential factors in the prediction model. In this study, serum oncological indicators such as CA125 and CEA were included as categorical variables. The cutoff value was set according to the laboratory diagnostic criteria, and the cutoff values for CA125, CEA, TPSA and CA153 were 35 U/ml, 5 ug/L, 80 U/L, and 25 U/ml, respectively.

To validate the performance of the prediction model, the construction cohort was first randomly divided into two sets: the training set (70%) and the test set (30%, internal validation). Additionally, 36 BC patients diagnosed between January 2011 and December 2015 acquired from the Russian Federation N.N.Blokhin National Medical Research Center of Oncology were included as the external validation cohort to test the generalizability of the model. Among these patients, 18 with DM (female; age: 33-73 years) and 18 without DM (female; age: 28-75 years) had complete MRI and clinicopathological records.

### MRI technique

Magnetic resonance images were acquired at the Tianjin Medical University Cancer Institute and Hospital using scanners manufactured by two different companies. MRI was performed with a 1.5-T system equipped with a dedicated four-channel phased-array bilateral breast coil (Signa Infinity Excite II, GE Healthcare) before 2013, while a 3.0-T MRI system equipped with a dedicated eight-channel phased-array breast coil (Discovery MR750, GE Medical Systems) was used after 2013. Some examinations were performed with a 3.0-T scanner using a dedicated 8-channel (4-channel for 1.5T scanner) phased-array breast coil. MRI protocols included axial T1-weighted imaging, fat-saturated fast spin-echo (FSE) sequences for T2-weighted imaging (T2WI) and unilateral sagittal fat-saturated FSE T2-weighted imaging of the affected breast before contrast administration. Diffusion-weighted imaging (DWI) was performed using a multi-section spin-echo single-shot echo-planar sequence bilaterally in the axial plane and in the sagittal plane of the affected breast. Images and sagittal data were obtained by sagittal DCE-MRI using the volume imaging for breast assessment (VIBRANT) bilateral breast imaging technique. Before the injection of the contrast agent, serial mask images were obtained. A contrast agent (Gd-DTPA, 0.2 mL/kg body weight, flow rate 2.0 mL/s) was manually injected using an automatic MR-compatible power injector and then flushed with the same total dose of saline solution. Dynamic MRI was immediately performed after the injection. Image acquisition was repeated five times (eight times for the 1.5T scanner), and each phase took 90-100 seconds (58-62 seconds for the 1.5T scanner). In all patients, final axial 3D fast spoiled gradient-recalled echo images were obtained after the dynamic study.

Magnetic resonance images in the N.N.Blokhin National Medical Research Center of Oncology were acquired through a 1.5-T MRI system equipped with the same four-channel phased-array bilateral breast coil. MRI protocols included axial T1-weighted imaging, fat-saturated fast spin-echo (FSE) sequences for T2-weighted imaging (T2WI) and unilateral sagittal fat-saturated FSE T2-weighted imaging of the affected breast before contrast administration. DWI was performed with the method. The image acquisition was repeated eight times, and each phase took 60-64 seconds.

### MRI analysis and postprocessing

Advantage Workstation AW 4.2 equipped with Functool II software (GE Healthcare) was employed for image postprocessing. A series of features, including lesion type, fibroglandular tissue, multiple masses, internal enhancement characteristics, signal intensity (compared with that of normal fibroglandular tissue of the breast) on T1- and T2-weighted images, background parenchymal enhancement and time-signal intensity curve (TIC) patterns, were analysed. To minimize the noise produced by the associated background, a limited region of interest was set within the lesion site. For the analysis of DCE-MR images, the evolution of the enhancement pattern at the periphery and in the centre of the tumour was recorded. TICs were classified as follows: type 1, slow or rapid initial contrast enhancement with a persistent delayed phase; type 2, rapid initial enhancement followed by a plateau in signal intensity; and type 3, rapid initial enhancement followed by rapid washout.

The MRI findings were independently analysed by two experienced breast radiologists with a minimum of 5 years of working experience. The readers interpreted the MR images independently using the 2013 MRI Breast Imaging Reporting and Data System (BI-RADS) tool from the American College of Radiology [21]. Differences in interpretation were resolved by reviewing and discussing the images according to the BI-RADS standard.

### Statistical analysis

Continuous variables are presented as the mean (±standard deviation), and categorical variables are presented as numbers and percentages. Differences in continuous variables were analysed with Student’s t-tests, and differences in categorical variables were tested with the chi-square test, Fisher’s exact test or Wilcoxon sum-rank test. Features with significant differences (*p* < 0.05) between BC patients with and without metastasis were further analysed by using the least absolute shrinkage and selection operator (LASSO) method to select the optimal subset based on the binomial deviance minimization criteria. Based on the aforementioned factors, a multivariate logistic regression model was adopted to establish two nomograms for predicting the risk of DM in BC: clinicopathological-feature alone model vs multi-feature fusion model. The performance of the nomogram was evaluated by diagnostic accuracy, sensitivity, specificity, area under the receiver operating characteristic (ROC) curve and calibration curves. The regression smoothing method was used to produce the calibration plots by bootstrapping with 1,000 resamples, where the relationship between the observed and predicted probabilities of DM was described graphically. The difference in the area under the curve (AUC) between the training and validation datasets was tested by the P-value of Integrated Discrimination Improvement (IDI) and Delong’s test. The validity and accuracy of the proposed models were further tested by the external validation cohort.

The diagnostic accuracy was calculated as (true positive+ true negative)/(true positive + false positive + false positive + true negative)× 100%. The 95% CIs of sensitivity, specificity and diagnostic accuracy were calculated with the website http://vassarstats.net/clin1.html. Other statistical analyses were conducted using R software (version 6.1, R Foundation for Statistical Computing, Vienna, Austria). A two-tailed difference with *p*<0.05 was considered significant.

## Supplementary Material

Supplementary Table 1

Supplementary Table 2
