# Supplementary material for: Distant metastasis prediction via a multi-feature fusion model in breast cancer
Source: Aging (Albany NY). 2020 Sep 28;12(18):18151–62. doi: 10.18632/aging.103630 (PMC7585122; doi:10.18632/aging.103630)
Supplement: Supplementary Table 1 [file aging-12-103630-s001..docx]

**Supplementary Table 1. Baseline demographic and clinicopathologic and MR imaging characteristics in the present cohort and external validation cohort.**

| **Character** | **Model development** | | | **Internal validation** | | | | | **External validation** | |
| --- | --- | --- | --- | --- | --- | --- | --- | --- | --- | --- |
|  | **Construction cohort**  **(N=201, 100%)** | | | **Training set**  **(N=141, 70%)** | | | **Test set  (N=60, 30%)** | | **Validation cohort  (N=36, 100%)** | |
|  | **Distant metastasis**  **(N, %)** | **Non-distant metastasis**  **(N, %)** | | **Distant metastasis**  **(N, %)** | **Non-distant metastasis**  **(N, %)** | | **Distant metastasis**  **(N, %)** | **Non-distant metastasis**  **(N, %)** | **Distant metastasis**  **(N, %)** | **Non-distant metastasis**  **(N, %)** |
| **Demographic and Clinic pathologic characteristics** | | | | | | | | | | |
| Age (mean±SD, years) | 47.13±11.93 | 47.16±9.67 | 47.55±12.3 | | 47.58±9.87 | 46.09±10.70 | | 46.18±8.59 | 56.55±12.62 | 50.00±10.72 |
| Family history of BC |  |  |  | |  |  | |  |  |  |
| Yes | 3 (4.48) | 8 (5.97) | 2 (4.44 ) | | 6 (6.25) | 1 (4.55) | | 2 (5.26) | 3 (16.67) | 0 (0.00) |
| No | 64 (95.52) | 126 (94.03) | 43 (95.56) | | 90 (93.75) | 21 (95.45) | | 36 (94.74) | 15 (83.33) | 18 (100.00) |
| Breast-feeding histories |  |  |  | |  |  | |  |  |  |
| Yes | 53 (79.10) | 117 (87.31) | 37 (82.22) | | 86 (89.58) | 16 (72.73) | | 31 (81.58) | 18 (100.00) | 16 (88.89) |
| No | 14(20.90) | 17 (12.69) | 8 (17.78) | | 10 (10.42) | 6 (27.27) | | 7 (18.42) | 0 (0.00) | 2 (11.11) |
| Marital status |  |  |  | |  |  | |  |  |  |
| Married | 63 (94.03) | 132 (98.51) | 42 (93.33) | | 95 (98.96) | 21 (4.55) | | 37 (97.37) | 18 (100.00) | 17 (94.44) |
| Never married | 4 (5.97) | 2 (1.49) | 3 (6.67) | | 1 (1.04) | 1(95.45) | | 1 (2.63) | 0 (0.00) | 1 (5.56) |
| Abortion |  |  |  | |  |  | |  |  |  |
| Yes | 34 (50.75) | 81 (60.45) | 22 (48.89) | | 55 (57.59) | 12 (54.55) | | 26 (68.42) | 0 (0.00) | 0 (0.00) |
| No | 33 (49.25) | 53(39.55) | 23 (51.11) | | 41 (42.71) | 10 (45.45) | | 12 (31.58) | 18 (100.00) | 18 (100.00) |
| The numbers of abortion |  |  |  | |  |  | |  |  |  |
| 0 | 33 (49.25) | 53 (39.55) | 23 (51.11) | | 41 (42.71) | 10 (45.45) | | 12 (31.58) | 18 (100.00) | 18 (100.00) |
| 1 | 13 (19.41) | 39 (29.10) | 9 (20.00) | | 30 (31.25) | 4 (18.18) | | 9 (23.68) | 0 (0.00) | 0 (0.00) |
| 2 | 16(23.88) | 26 (19.40) | 11 (24.44) | | 15 (15.62) | 5 (22.73) | | 11 (28.95) | 0 (0.00) | 0 (0.00) |
| >2 | 5 (7.46) | 16 (11.95) | 2 (0.00) | | 10 (10.38) | 3 (13.59) | | 6 (15.83) | 0 (0.00) | 0 (0.00) |
| Reproductive history |  |  |  | |  |  | |  |  |  |
| Yes | 57 (85.07) | 128 (95.52) | 38 (84.44) | | 93 (96.88) | 19 (86.36) | | 35 (92.11) | 18 (100.00) | 17 (94.44) |
| No | 10 (14.93) | 6 (4.48) | 7 (15.56) | | 3 (3.12) | 3 (13.64) | | 3 (7.89) | 0 (0.00) | 1 (5.56) |
| Parity |  |  |  | |  |  | |  |  |  |
| 0 | 10 (14.93) | 6 (4.48) | 7 (15.56) | | 3 (3.12) | 3 (13.64) | | 3 (7.89) | 0 (0.00) | 1 (5.56) |
| 1 | 33 (49.25) | 105 (78.36) | 21 (46.67) | | 75 (78.13) | 12 (54.55) | | 30 (78.95) | 11 (61.11) | 14 (77.78) |
| 2 | 17 (25.37) | 21 (15.67) | 10 (22.22) | | 17 (17.71) | 7 (31.81) | | 4 (10.53) | 6 (33.33) | 3 (16.66) |
| >2 | 7 (10.45) | 2 (1.49) | 7 (15.55) | | 1 (1.04) | 0 (0.00) | | 1 (2.63) | 1 (5.56) | 0 (0.00) |
| Menstrual status |  |  |  | |  |  | |  |  |  |
| Menstruate 0 | 40 (59.70) | 88 (65.67) | 26 (57.78) | | 63 (65.62) | 14 (63.64) | | 25 (65.79) | 6 (33.33) | 12 (66.67) |
| Menopause 1 | 27 (40.30) | 46 (34.33) | 19 (42.22) | | 33 (34.38) | 8 (36.36) | | 13 (34.21) | 12 (66.67) | 6 (33.33) |
| Age of menarche | 14.35 ±1.89 | 14.44 ±1.74 | 14.20±1.90 | | 14.55±1.79 | 14.77±1.85 | | 14.11±1.56 | 12.94 ±1.16 | 14.11±1.937 |
| Lymph node metastasis |  |  |  | |  |  | |  |  |  |
| None | 27 (40.30) | 101 (75.37) | 16 (35.56) | | 75 (78.12) | 11 (50.00) | | 26 (68.42) | 8 (44.44) | 14 (77.78) |
| Have | 40 (59.70) | 33 (24.63) | 29 (64.44) | | 21 (21.88) | 11 (50.00) | | 12 (31.58) | 10 (55.56) | 4 (22.22) |
| ER |  |  |  | |  |  | |  |  |  |
| Positive | 41 (61.19) | 107 (79.85) | 26 (57.78) | | 75 (78.12) | 15 (68.18) | | 32 (84.21) | 10 (55.56) | 17 (94.44) |
| Negative | 26 (38.81) | 27 (20.15) | 19 (42.22) | | 21 (21.88) | 7 (31.82) | | 6 (15.79) | 8 (44.44) | 1 (5.56) |
| PR |  |  |  | |  |  | |  |  |  |
| Positive | 38 (56.72) | 104 (77.61) | 24 (53.33) | | 74 (77.08) | 14 (63.64) | | 30 (78.95) | 10 (55.56) | 11 (61.11) |
| Negative | 29 (43.28) | 30 (22.39) | 21 (46.67) | | 22 (22.92) | 8 (36.36) | | 8 (21.05) | 8 (44.44) | 7 (38.89) |
| HER2 status |  |  |  | |  |  | |  |  |  |
| Positive | 28 (41.79) | 45 (33.58) | 20 (44.44) | | 37 (38.54) | 8 (36.36) | | 8 (21.05) | 9 (50.00) | 8 (44.44) |
| Negative | 39 (58.21) | 89 (66.42) | 25 (55.56) | | 59 (61.46) | 14 (63.64) | | 30 (78.95) | 9 (50.00) | 10 (55.56) |
| Ki-67 |  |  |  | |  |  | |  |  |  |
| Positive | 56 (83.58) | 118 (88.06) | 38 (84.44) | | 87 (90.62) | 18 (81.82) | | 31 (81.58) | 12 (66.67) | 15 (83.33) |
| Negative | 11 (16.42) | 16 (11.94) | 7 (15.56) | | 9 (9.38) | 4 (18.18) | | 7 (18.42) | 6 (33.33) | 3 (16.67) |
| TPSA |  |  |  | |  |  | |  |  |  |
| Positive | 9 (13.43) | 14(10.45) | 7 (15.56) | | 11 (11.64) | 2 (9.09) | | 3 (7.89) | 2 (11.11) | 2 (11.11) |
| Negative | 58 (86.57) | 120(89.55) | 38 (84.44) | | 85 (85.54) | 20 (90.91) | | 35 (92.11) | 16 (88.89) | 16 (88.89) |
| CA153 |  |  |  | |  |  | |  |  |  |
| Positive | 17 (25.37) | 1 (0.75) | 13 (28.89) | | 1 (1.04) | 4 (18.18) | | 0 (0.00) | 3 (16.67) | 1 (5.56) |
| Negative | 50 (74.63) | 133 (99.25) | 32 (71.11) | | 95 (98.96) | 18 (81.82) | | 38 (100.00) | 15 (83.33) | 17 (94.44) |
| CEA |  |  |  | |  |  | |  |  |  |
| Positive | 13 (19.40) | 1 (0.75) | 11 (24.44) | | 0 (0.00) | 2 (9.09) | | 1 (2.63) | 3 (16.67) | 1 (5.56) |
| Negative | 54 (80.60) | 133 (99.25) | 34 (75.56) | | 96 (100.00) | 20 (90.91) | | 37 (97.37) | 15 (83.33) | 17 (94.44) |
| CA125 |  |  |  | |  |  | |  |  |  |
| Positive | 11 (16.42) | 8 (5.97) | 7 (15.56) | | 5 (5.21) | 4 (18.18) | | 3 (7.89) | 2 (11.11) | 1 (5.56) |
| Negative | 56 (83.58) | 126 (94.03) | 38 (84.44) | | 91 (94.79) | 18 (81.82) | | 35 (92.11) | 16 (88.89) | 17 (94.44) |
| Operation |  |  |  | |  |  | |  |  |  |
| No surgery | 7 (10.45) | 0 (0.00) | 5 (11.11) | | 0 (0.00) | 2 (9.09) | | 0 (0.00) | 1 (5.56) | 0 (00.00) |
| Conserving | 16 (23.88) | 58 (43.28) | 12 (26.67) | | 46 (47.92) | 4 (18.18) | | 12 (31.58) | 7 (38.89) | 14 (77.78) |
| Radical | 44 (65.67) | 76 (56.72) | 28 (62.22) | | 50 (52.08) | 16 (72.73) | | 26 (68.42) | 10 (55.55) | 4 (22.22) |
| Endocrinotherapy |  |  |  | |  |  | |  |  |  |
| Yes | 0 (0.00) | 12 (8.96) | 0 (0.00) | | 7 (7.29) | 0 (0.00) | | 5 (13.16) | 0 (0.00) | 1 (5.56) |
| No | 67 (100.00) | 122 (91.04) | 45 (100.00) | | 89 (92.71) | 22 (100.00) | | 33 (86.84) | 18 (100.00) | 17 (94.44) |
| Radiotherapy |  |  |  | |  |  | |  |  |  |
| Yes | 14 (20.90) | 17 (12.69) | 10 (22.22) | | 10 (10.42) | 4 (18.18) | | 7 (18.42) | 13 (72.22) | 17 (94.44) |
| No | 53 (79.10) | 117 (87.31) | 35 (77.78) | | 86 (89.58) | 18 (81.82) | | 31 (81.58) | 5 (27.28) | 1 (5.56) |
| Chemotherapy |  |  |  | |  |  | |  |  |  |
| Yes | 66 (98.51) | 120 (89.55) | 45 (100.00) | | 85 (88.54) | 21 (95.45) | | 35 (92.11) | 16 (88.89) | 16 (88.89) |
| No | 1 (1.49) | 14 (10.45) | 0 (0.00) | | 11 (11.46) | 1 (4.55) | | 3 (7.89) | 2 (11.11) | 2 (11.11) |
| **MR imaging characteristics** | | | | | | | | | | |
| Multiple mass |  |  |  | |  |  | |  |  |  |
| Yes | 23 (34.33) | 9 (6.72) | 17 (37.78) | | 7 (7.29) | 6 (27.27) | | 2 (5.26) | 10 (55.56) | 3 (16.67) |
| No | 44 (65.67) | 125 (93.28) | 28 (62.22) | | 89 (92.71) | 16 (72.73) | | 36 (94.74) | 8 (44.44) | 15 (83.33) |
| Internal enhancement |  |  |  | |  |  | |  |  |  |
| Homogeneous | 9 (13.43) | 25 (18.66) | 7 (15.56) | | 15 (15.62) | 2 (90.9) | | 10 (26.32) | 2 (11.11) | 6 (33.33) |
| Heterogeneous | 58 (86.57) | 109 (81.34) | 38 (84.44) | | 81 (84.38) | 20 (90.91) | | 28 (73.68) | 16 (88.89) | 12 (66.67) |
| T1 WI signal |  |  |  | |  |  | |  |  |  |
| Homogeneous | 59 (88.06) | 131 (97.76) | 41 (91.11) | | 94 (97.92) | 18 (81.82) | | 37 (97.37) | 13 (72.22) | 17 (94.44) |
| Heterogeneous | 8 (11.94) | 3 (2.24) | 4 (8.89) | | 2 (2.08) | 4 (18.18) | | 1 (2.63) | 5 (27.78) | 1 (5.56) |
| Fat-saturated T2 WI signal |  |  |  | |  |  | |  |  |  |
| Homogeneous | 31 (46.27) | 82 (61.19) | 20 (44.44) | | 57 (59.38) | 11 (50.00) | | 25 (65.79) | 6 (33.33) | 14 (77.78) |
| Heterogeneous | 36 (53.73) | 52 (38.81) | 25 (55.56) | | 39 (40.62) | 11 (50.00) | | 13 (34.21) | 12 (66.67) | 4 (22.22) |
| Kinetic curve pattern |  |  |  | |  |  | |  |  |  |
| Type 1 | 9 (13.43) | 19 (14.18) | 5 (11.11) | | 15 (15.63) | 4 (18.18) | | 4 (10.53) | 3 (16.67) | 5 (27.78) |
| Type 2 | 5 (7.46) | 5 (3.73) | 4 (8.89) | | 3 (3.12) | 1 (4.55) | | 2 (5.26) | 3 (16.67) | 0 (0.00) |
| Type 3 | 53 (79.11) | 110 (82.09) | 36 (80.00) | | 78 (81.25) | 17 (77.27) | | 32 (84.21) | 12 (66.66) | 13 (72.22) |
| Parenchymal enhancement |  |  |  | |  |  | |  |  |  |
| Minimal | 14 (20.90) | 29 (21.64) | 9 (20.00) | | 22 (22.92) | 5 (22.73) | | 7 (18.42) | 3 (16.67) | 4 (22.22) |
| Mild | 27 (40.30) | 67 (50.00) | 16 (35.56) | | 48 (50.00) | 11 (50.00) | | 19 (50.00) | 7 (38.88) | 9 (50.00) |
| Moderate | 17 (25.37) | 29 (21.64) | 13 (28.89) | | 23 (23.96) | 4 (18.18) | | 6 (15.79) | 5 (27.78) | 3 (16.67) |
| Marked | 9 (13.43) | 9 (6.72) | 7 (15.56) | | 3 (3.12) | 2 (9.09) | | 6 (15.79) | 3 (16.67) | 2 (11.11) |
| Fibroglandular tissue |  |  |  | |  |  | |  |  |  |
| Fatty | 1 (1.49) | 5 (3.73) | 0 (0.00) | | 4 (4.17) | 1 (4.55) | | 1 (2.63) | 6 (33.33) | 5 (27.78) |
| Scattered | 11 (16.42) | 19 (14.18) | 7 (15.56) | | 15 (15.62) | 4 (18.18) | | 4 (10.53) | 3 (16.67) | 3 (16.67) |
| Heterogeneous | 48 (71.64) | 99 (73.88) | 32 (71.11) | | 68 (70.83) | 16 (72.73) | | 31 (81.58) | 5 (27.78) | 7 (38.88) |
| Extremely dense | 7 (10.45) | 11 (8.21) | 6 (13.33) | | 9 (9.38) | 1 (4.55) | | 2 (5.26) | 4 (22.22) | 3 (16.67) |
| Lesion size |  |  |  | |  |  | |  |  |  |
| ≤2 | 7 (10.45) | 58 (43.28) | 5 (11.11) | | 42 (43.75) | 2 (9.09) | | 16 (42.11) | 3 (16.67) | 4 (22.22) |
| >2 and ≤5 | 36 (53.73) | 63 (47.02) | 23 (51.11) | | 46 (47.92) | 13 (59.09) | | 17 (44.74) | 10 (55.56) | 11 (61.11) |
| >5 | 24 (35.82) | 13 (9.70) | 17 (37.78) | | 8 (8.33) | 7 (31.82) | | 5 (13.16) | 5 (27.77) | 3 (16.67) |
| Lesion type |  |  |  | |  |  | |  |  |  |
| Masses | 40 (59.70) | 108 (80.60) | 27 (60.00) | | 79 (82.29) | 13 (59.09) | | 29 (76.32) | 13 (72.22) | 15 (83.33) |
| Nonmass | 18 (26.87) | 18 (13.43) | 11 (24.44) | | 12 (12.50) | 7 (31.82) | | 6 (15.79) | 3 (16.67) | 1 (5.56) |
| Mixed | 9 (13.43) | 8 (5.97) | 7 (15.56) | | 5 (5.21) | 2 (9.09) | | 3 (7.89) | 2 (11.11) | 2 (11.11) |

**Abbreviations:** ER: Expression of the oestrogen receptor; PR: Progesterone receptor; HER2: Human epidermal growth factor receptor 2; TPSA: Total prostate-specific antigen; CA125: Carbohydrate antigen 125; CEA: Carcinoembryonic antigen; CA153: Carbohydrate antigen 153.
