# Supplementary material for: Distant metastasis prediction via a multi-feature fusion model in breast cancer
Source: Aging (Albany NY). 2020 Sep 28;12(18):18151–62. doi: 10.18632/aging.103630 (PMC7585122; doi:10.18632/aging.103630)
Supplement: Supplementary Table 2 [file aging-12-103630-s002..docx]

**Supplementary Table 2. Differences in the clinicopathologic and MR imaging characteristics between breast cancer patients with and without distant metastasis in the construction cohort.**

| **Character** | **Distant metastasis** | | ***χ^2^ /Z*** | ***P*-value** |
| --- | --- | --- | --- | --- |
|  | **Yes (N, %)** | **No (N, %)** |  |  |
| Age (mean±SD, years) | 47.13±11.93 | 47.16±9.67 | -0.014 | 0.989 |
| Family history of BC |  |  | 0.192 | 0.661 |
| Yes | 3 (4.48) | 8 (5.97) |  |  |
| No | 64 (95.52) | 126 (94.03) |  |  |
| Breast-feeding histories |  |  | 2.308 | 0.129 |
| Yes | 53 (79.10%) | 117 (87.31%) |  |  |
| No | 14(20.90%) | 17 (12.69%) |  |  |
| Marital status |  |  | 3.092 | 0.079 |
| Married | 63 (94.03%) | 132 (98.51%) |  |  |
| Never married | 4 (5.97%) | 2 (1.49%) |  |  |
| Abortion |  |  | 1.717 | 0.19 |
| Yes | 34 (50.75%) | 81 (60.45%) |  |  |
| No | 33 (49.25%) | 53(39.55%) |  |  |
| The numbers of abortion |  |  | 3.893 | 0.273 |
| 0 | 33 (49.25%) | 53 (39.55) |  |  |
| 1 | 13 (19.41%) | 39 (29.10) |  |  |
| 2 | 16(23.88%) | 26 (19.40) |  |  |
| >2 | 5 (7.46%) | 16 (11.95) |  |  |
| Reproductive history |  |  | 6.655 | 0.010 |
| Yes | 57 (85.07) | 128 (95.52) |  |  |
| No | 10 (14.93) | 6 (4.48) |  |  |
| Parity |  |  | 21.860 | <0.001 |
| 0 | 10 (14.93) | 6 (4.48) |  |  |
| 1 | 33 (49.25) | 105 (78.36) |  |  |
| 2 | 17 (25.37) | 21 (15.67) |  |  |
| >2 | 7 (10.45) | 2 (1.49) |  |  |
| Menstrual status |  |  | 0.688 | 0.407 |
| Menstruate | 40 (59.70) | 88 (65.67) |  |  |
| Menopause | 27 (40.30) | 46 (34.33) |  |  |
| Age of menarche | 14.354 (1.89) | 14.440 (1.74) | -0.136 | 0.892 |
| Lymph node metastasis |  |  | 23.759 | <0.001 |
| None | 27 (40.30) | 101 (75.37) |  |  |
| Have | 40 (59.70) | 33 (24.63) |  |  |
| ER |  |  | 8.008 | 0.005 |
| Positive | 41 (61.19) | 107 (79.85) |  |  |
| Negative | 26 (38.81) | 27 (20.15) |  |  |
| PR |  |  | 9.405 | 0.002 |
| Positive | 38 (56.72) | 104 (77.61) |  |  |
| Negative | 29 (43.28) | 30 (22.39) |  |  |
| HER2 status |  |  | 1.301 | 0.254 |
| Positive | 28 (41.79) | 45 (33.58) |  |  |
| Negative | 39 (58.21) | 89 (66.42) |  |  |
| Ki-67 |  |  |  |  |
| Positive | 56 (83.58) | 118 (88.06) | 0.770 | 0.382 |
| Negative | 11 (16.42) | 16 (11.94) |  |  |
| TPSA |  |  | 0.393 | 0.531 |
| Positive | 9 (13.43) | 14(10.45) |  |  |
| Negative | 58 (86.57) | 120(89.55) |  |  |
| CA153 |  |  | 33.225 | <0.001 |
| Positive | 17 (25.37) | 1 (0.75) |  |  |
| Negative | 50 (74.63) | 133 (99.25) |  |  |
| CEA |  |  | 23.993 | <0.001 |
| Positive | 13 (19.40) | 1 (0.75) |  |  |
| Negative | 54 (80.60) | 133 (99.25) |  |  |
| CA125 |  |  | 5.696 | 0.017 |
| Positive | 11 (16.42) | 8 (5.97) |  |  |
| Negative | 56 (83.58) | 126 (94.03) |  |  |
| Operation |  |  | 19.168 | <0.001 |
| No surgery | 7 (10.45) | 0 (0.00) |  |  |
| Conserving | 16 (23.88) | 58 (43.28) |  |  |
| Radical | 44 (65.67) | 76 (56.72) |  |  |
| Endocrinotherapy |  |  | 6.381 | 0.011 |
| Yes | 0 (0.00) | 12 (8.96) |  |  |
| No | 67 (100.00) | 122 (91.04) |  |  |
| Radiotherapy |  |  | 2.308 | 0.129 |
| Yes | 14 (20.90) | 17 (12.69) |  |  |
| No | 53 (79.10) | 117 (87.31) |  |  |
| Chemotherapy |  |  |  |  |
| Yes | 66 (98.51) | 120 (90.30) | 3.395 | 0.065 |
| No | 1 (1.49) | 14 (10.40) |  |  |
| Multiple mass |  |  | 25.441 | <0.001 |
| Yes | 23 (34.33) | 9 (6.72) |  |  |
| No | 44 (65.67) | 125 (93.28) |  |  |
| Internal enhancement |  |  | 2.571 | 0.109 |
| Homogeneous | 9 (13.43) | 25 (18.66) |  |  |
| Heterogeneous | 58 (86.57) | 109 (81.34) |  |  |
| T1 WI signal |  |  | 8.127 | 0.004 |
| Homogeneous | 59 (88.06) | 131 (97.76) |  |  |
| Heterogeneous | 8 (11.94) | 3 (2.24) |  |  |
| Fat-saturated T2 WI signal |  |  | 4.043 | 0.044 |
| Homogeneous | 31 (46.27) | 82 (61.19) |  |  |
| Heterogeneous | 36 (53.73) | 52 (38.81) |  |  |
| Kinetic curve pattern |  |  | 1.132 | 0.517 |
| Type 1 | 9 (13.43) | 19 (14.18) |  |  |
| Type 2 | 5 (7.46) | 5 (3.73) |  |  |
| Type 3 | 53 (79.11) | 110 (82.09) |  |  |
| Parenchymal enhancement |  |  | 3.432 | 0.330 |
| Minimal | 14 (20.90) | 29 (21.64) |  |  |
| Mild | 27 (40.30) | 67 (50.00) |  |  |
| Moderate | 17 (25.37) | 29 (21.64) |  |  |
| Marked | 9 (13.43) | 9 (6.72) |  |  |
| Fibroglandular tissue |  |  | 1.181 | 0.758 |
| Fatty | 1 (1.49) | 5 (3.73) |  |  |
| Scattered | 11 (16.42) | 19 (14.18) |  |  |
| Heterogeneous | 48 (71.64) | 99 (73.88) |  |  |
| Extremely dense | 7 (10.45) | 11 (8.21) |  |  |
| Lesion size |  |  | 31.855 | <0.001 |
| ≤2 | 7 (10.45) | 58 (43.28) |  |  |
| >2 and ≤5 | 36 (53.73) | 63 (47.02) |  |  |
| >5 | 24 (35.82) | 13 (9.70) |  |  |
| Lesion type |  |  | 10.090 | 0.006 |
| Masses | 40 (59.70) | 108 (80.60) |  |  |
| Nonmass | 18 (26.87) | 18 (13.43) |  |  |
| Mixed | 9 (13.43) | 8 (5.97) |  |  |

**Abbreviations:** ER: Estrogen receptor; PR: Progesterone receptor; HER2: Human epidermal growth factor receptor 2; PSA: Prostate-specific antigen; CA125: Carbohydrate antigen 125; CEA: Carcinoembryonic antigen; CA153: Carbohydrate antigen 153.
